# Formation and Reactions of Brønsted and Lewis Acid Adducts with Electron-Rich Heteroaromatic Compounds

**DOI:** 10.3390/molecules29133151

**Published:** 2024-07-02

**Authors:** Horst Hartmann, Jürgen Liebscher

**Affiliations:** 1 Fakultät Chemie und Lebensmittelchemie, Technische Universität Dresden, 01069 Dresden, Germany; 2 National Institute for Research and Development of Isotopic and Molecular Technologies INCDTIM, 400293 Cluj-Napoca, Romania; liebscher@chemie.hu-berlin.de; 3 Institute of Chemistry, Humboldt-University of Berlin, 12489 Berlin, Germany

**Keywords:** furan, thiophene, pyrrole, protonation, deuteration, Brønsted acid, Lewis acid, mercuration

## Abstract

Electron-rich heteroaromatics, such as furan, thiophene and pyrrole, as well as their benzo-condensed derivatives, are of great interest as components of natural products and as starting substances for various products including high-tech materials. Although their reactions with Brønsted and Lewis acids play important roles, in particular as the primary step of various transformations, they are often disregarded and mechanistically not understood. The present publication gives a first overview about this chemistry focusing on the parent compounds. It comprises reactions with strong Brønsted acids forming adducts that can undergo intramolecular proton and/or substituent transfer reactions, ring openings or ring transformations into other heterocycles, depending on their structure. Interactions with weak Brønsted acids usually initiate oligomerizations/polymerizations. A similar behaviour is observed in reactions of these heteroaromatics with Lewis acids. Special effects are achieved when the Lewis acids are activated through primary protonation. Deuterated Brønsted acids allow straight forward deuteration of electron-rich heteroaromatics. Mercury salts as extremely weak Lewis acids cause direct metalation in a straight forward way replacing ring H-atoms yielding organomercury heterocycles. This review will provide comprehensive information about the chemistry of adducts of such heterocycles with Brønsted and Lewis acids enabling chemists to understand the mechanisms and the potential of this field and to apply the findings in future syntheses.

## 1. Introduction

The presence of Brønsted acids in reactions of aromatic or heteroaromatic compounds, such as electrophilic substitution, rearrangement or oligomerisation, is often considered as a mere catalytic effect and thus is not investigated in detail. However, detailed knowledge on the mode of interaction of the aromatic compounds with such acids is important, because it allows finding suitable Brønsted acids to optimize reaction conditions and to control deuteration reactions via treatment with deuterated Brønsted acids. Furthermore, the interaction of Brønsted acids with heteroaromatic compounds can cause interesting subsequent reactions, such as additions, oligomerizations, rearrangements and substituent migration. Herein, the first review is presented, summarizing the reaction of aza-free electron-rich heteroaromatic compounds with Brønsted acids, their deuterium analogues and with Lewis acids, comprising recent results from our laboratory. It is focused on the parent heteroaromatic systems and simple derivatives. Occasionally, a few simple non-heterocyclic aromatics are included, while compounds with more complicated substitution patterns or with aza moieties in their ring are not considered.

## 2. General Remarks on Electrophilic Substitution in Heteroaromatic Compounds

**Scheme 1:**
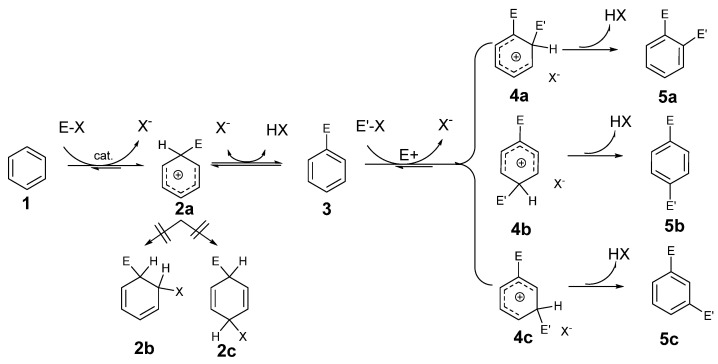
Reaction pathways in the reaction of benzene (**1**) with electrophilic reagents E-X.

It is well known and reported in common monographs and organic chemistry [1] textbooks that benzene (**1**), as a prototype of aromatic compounds, is able to react with electrophilic reagents of the general formula E-X, usually after activation of the corresponding electrophilic species with a suitable catalyst (Scheme 1) [2,3]. These catalysts, usually Brønsted or Lewis acids, release the more reactive species E^+^ from E-X and initiate the formation of an adduct **2a**, in which the electrophile is covalently bounded to the benzene ring. Subsequently, from this adduct **2a**, usually named the Wheland intermediate [4], the corresponding substitution product **3** is formed through loss of the proton at the ipso position. The introduction of the electrophilic moiety E into the benzene ring increases its reactivity towards electrophiles, e.g., if E = F, Cl, Br or alkyl, further substitution can occur with a second electrophile E’-X. Thereby, *ortho-* and *para-*disubstituted products **5a** and **5b** are obtained usually via **4a** and **4b**, respectively. In contrast, if the introduction of the electrophilic moiety **E** into the benzene ring decreases its reactivity towards the electrophiles, e.g., if E = NO_2_, SO_3_H, RCO or N = N-Aryl, a second substitution with a further electrophile E’-X usually gives rise to the formation of meta-substitution products **5c**. Evidently, these substitution products are generated via **4c**.

The ability of benzene and its derivatives to undergo substitution reactions (regenerative or meneïd reactivity [5]) instead of additions, which would yield products of the general structure **2b** or **2c**, are not favoured.

Nevertheless, there are some examples for addition reactions with benzene and some of its derivatives, such as the fluorination of benzene with AgF_2_, which gives rise to the formation of 3,6-difluoro-1,4-cyclohexadiene as the main product [6], or the chlorination of benzene with elemental chlorine, yielding different chloro-substituted cyclohexadienes, cyclohexenes and cyclohexanes [7]. Although a radical mechanism is widely assumed for these reactions, an electrophilic mechanism cannot be ruled out and has been discussed in the literature.

The occurrence of Wheland intermediate **2a** in the electrophilic substitution of aromatic compounds has not been unequivocally demonstrated in all reactions, because they are often short-lived intermediates that are rapidly converted to substitution products **3** [4]. However, since this transformation can also take place in a reversible direction, the formation of the Wheland intermediate **2a** should be favoured by increasing the proton concentration of the reaction mixture, e.g., by adding a strong Brønsted acid.

**Scheme 2:**
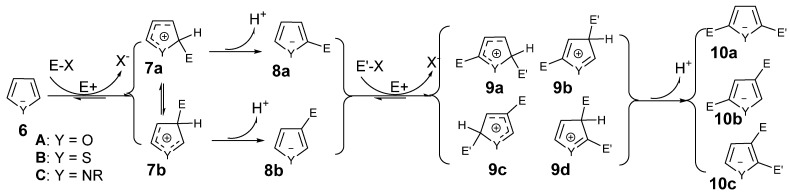
Reaction pathways in the reaction of heteroaromatics (**6**) with electrophilic reagents E-X.

As is well known, electrophilic substitution reactions can proceed in a similar way with hetero-analogues of benzene, such as furan (**6A**), thiophene (**6B**) and pyrrole (**6C**) and a variety of their derivatives (Scheme 2) [8]. Here, the electrophilic attack of the appropriate electrophile E-X can take place both at their 2- and 3-position, so that Wheland intermediates with isomeric structures **7a** and **7b** are formed, leading to two different substitution products **8a** and **8b** after deprotonation. Moreover, a repeated reaction with a further electrophile E’-X can occur with formation of di-substitution product **10a**–**10c** via intermediates **9a**–**9d** depending on the types of electrophiles used.

## 3. Reaction of Electron-Rich Heteroaromatic Compounds with Brønsted Acids

### 3.1. Formation and Properties of Proton Adducts

**Scheme 3:**
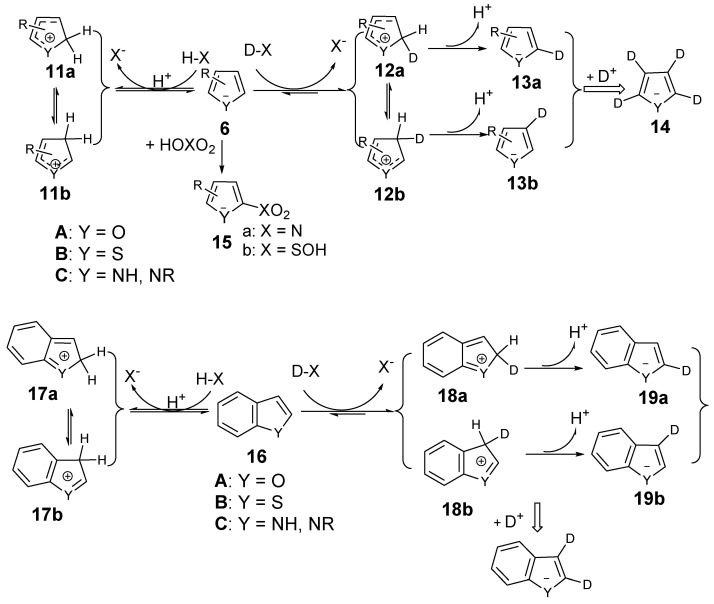
Mechanistic pathways in the reaction within the heterocyclic ring of heteroaromatics **6** and **16** with Brønsted acids and deuterated Brønsted acids.

Reactions of benzene (**1**) or its heterocyclic analogues **6A**–**6C** with sufficiently strong Brønstedt acids H-X can occur in a similar way to that observed with electrophilic reagents E-X. Whereas some mineral acids, such as nitric acid or sulphuric acid, act as electrophilic reagents in reactions with heterocyclic compounds **6** leading to corresponding nitro or sulfonic acid substitution products **15** [9,10] (Scheme 3), a variety of other Brønsted acids, such as phosphoric acid, perchloric acid, trifluoroactic acid (TFA), trifluoromethanesulphonic acid (TFS) or common Brønsted acids activated by acidic Zeolites [3] cannot produce substitution products **8** or **10** (Scheme 2) in this way. However, very strong Brønsted acids can reversibly protonate heterocycles **6** leading to cations of the general structure **11a** and **11b** (Scheme 3). This phenomenon is often not recognized because the equilibrium is predominantly at the side of starting materials and the reversibility of the protonation resulting in re-isolation of the starting compounds **6** after working up. Similar argumentations hold true for benzo-condensed heterocycles **16**.

If, however, strong Brønsted acids are used and the concentration of the formed proton adducts **11** is high enough, their identification with special spectroscopic methods, especially by means of ^1^H NMR spectroscopy, is possible. In this way, proton adducts **11** generated through treatment of the heterocycles **6** or some of their simple derivatives with super acids as well as with TFA or TFS have been unambiguously identified by means of ^1^H NMR measurements. For example, for thiophene (**6B**) and its 2-methyl, 3-methyl and 2,4-dimethyl derivatives, ^1^H NMR spectra recorded in HF and HBF_4_ indicate the corresponding proton adducts **11Ba** or **11Bb** [11]. Analogously, the proton adduct generated through treatment of 2-methylmercaptothiophene with TFS could be identified with ^1^H NMR spectroscopy [12]. For certain halothiophenes, ^1^H and ^13^C NMR measurements performed in FSO_3_H indicate unambiguously the formation of the C2 protonated species **11Ba** [13,14].

Appropriate proton adducts of the general structure **11Aa** were also identified for some *tert*-butyl-substituted furans [15] as well as for the products generated in a different way via treatment of 5-methyl-5-hexen-2-one with FSO_3_H/SbF_5_ [16,17].

According to ^1^H NMR measurements, pyrrole (**6C**) and some of its *C*- and *N*-substituted derivatives form definite proton adducts of the structure **11Ca** upon treatment with ethereal HBF_4_ [18]. In contrast, isomeric proton adducts **11Cb** appear in reaction of pyrrole (**6C**) with 16 N sulphuric acid or TFS as evidenced with ^1^H NMR spectroscopy [19,20,21,22,23,24,25,26,27,28,29,30]. Analogous results were obtained through treatment of *N*-aryl-substituted pyrroles with TFS resulting in **11Cb** (Y = NAryl) [31].

Pyrrolium salts of the structure **11Ca** have been indicated for some amino- and methylmercapto-substituted derivatives [32,33] as well as for a few 5-furyl-substituted pyrroles [34].

C2 protonated species **11Aa** and **11Ca** were generated from furan (**6A**) and pyrrole (**6C**) through chemical ionization MS spectrometry where CH_5_^+^ or C_2_H_5_^+^ served as protonating agents [35,36]. Proton adducts of furans **6A** could also be indicated as Wheland intermediates in the mass spectrometer by the reaction of the parent compounds with gaseous CH_3_^+^ [37]. Proton adducts of furans generated through HCl-catalysed cyclisation of 2,5-alkanediones have been detected with mass spectroscopy, while also estimating the relative stability of the isomers was possible [38].

By means of MO calculations, the relative stabilities of the proton adducts of the five-membered heterocyclic compounds **6** and some of their benzo-condensed derivatives **16** have been estimated [39,40,41]. For the same compounds **6,** the basicity constants have been calculated and related to the aromatic resonance energies [42].

Proton adducts have also been revealed for a variety of electron-rich benzo-condensed 5-ring heteroaromatics **16** (Scheme 3). Thus, definite proton adducts of the structure **17Cb** were observed after treatment of some indoles (**16C**) with conc. H_2_SO_4_ [43], phosphoric acid [44], trifluoracetic acid [45], HClO_4_ [46] or super acids [47]. In all cases, the proton is added at C3 of the indole giving rise to the formation of the proton adduct **17Cb**.

On the other hand, proton adducts of the structure **17b** (Y = S, O) derived from benzo[*b*]thiophene (**16B**) and benzo[*b*]furane (**16A**) have not been found by using strong Brønsted acids, but only in the flame ionization mass spectrometer [48,49]. However, structures of the proton adducts were not provided.

**Scheme 4:**
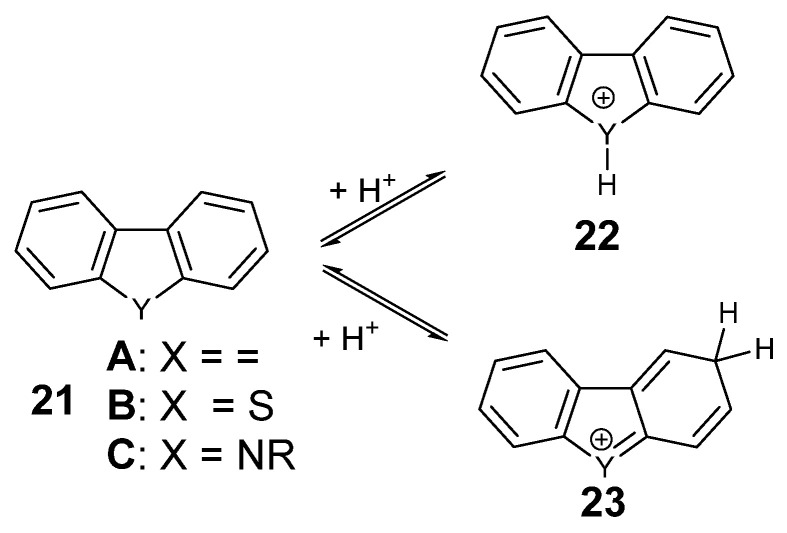
Reaction of heteroaromatics **21** with Brønsted acids.

Proton adducts are formed when derivatives of dibenzofuran (**21A**) [50] and dibenzothiophene (**21B**) [51], as well as carbazoles (**21C**), were treated with strong Brønsted or super acids. However, the protonation of the former compounds (**21A**) and (**21B**) does not occur at their heterocyclic moiety but at the benzo ring, giving rise to the formation of compounds (**23**), while *N*-aryl-substituted carbazoles **21C** undergo simultaneous protonation at their *N-*atom as well as at the *C*-atoms to form compounds **22C** and **23C** [52]. The *N-*unsubstituted and *N*-methylcarbazole **21C** are exclusively protonated at their *N*-atom under formation of compounds **22C** (Y = NR) (Scheme 4) [53].

With all the considerations about the formation of cationic proton adducts **11** of heterocycles in mind, one should be aware that they can undergo a rapid proton exchange between the C2 and C3 position in analogy to the proton exchange at the proton adduct of certain alkyl benzenes, such as of toluene or ethylbenzene [54]. In consequence, both species **11a** and **11b** (Scheme 3) can exist side by side simultaneously [26]. To clarify this problem, low-temperature measurements of proton adducts **11** seem to be necessary.

An alternative, powerful method for clarifying the proton exchange reaction at Wheland intermediates or the simultaneous formation of isomeric Wheland complexes consists in the use of deuterated Brønsted acids. Details on this method are discussed in the Section 3.2.3.

### 3.2. Properties of Proton Adducts of Simple Heteroaromatic Compounds

#### 3.2.1. Proton Migration

For the proton adducts of aromatic compounds, a quick proton exchange between adjacent positions was indicated through ^1^H NMR measurements. For example, in the hexamethylbenzenium cation, this proton exchange occurs very quickly at room temperature with a rate of 10^2^ s^−1^. However, it can be frozen at lower temperature and gives rise to sharp lines in the proton spectrum [55].

**Scheme 5:**
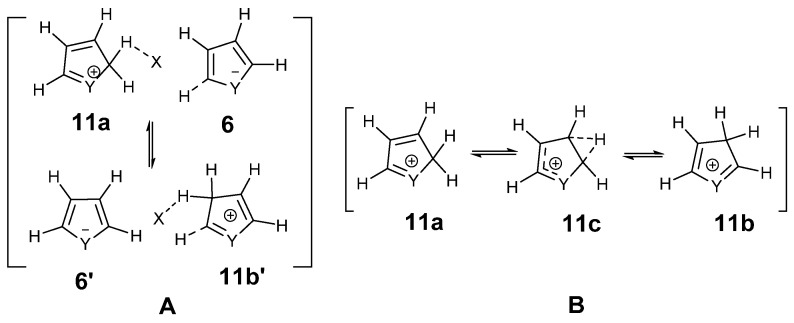
Alternative mechanism (**A**,**B**) of the proton migration at heteroaromatics **6.**

Consistent with this result, a proton exchange between different ring positions in the corresponding proton adducts of heteroaromatic compounds **6** takes place. Thus, the proton adducts (**11**) differ in the site of proton attachment resulting from disagreement between kinetic and thermodynamic stability [56,57]. This proton exchange can occur in different ways, e.g., through a (bimolecular) intermolecular exchange reaction between **11** and the parent heterocyclic component (**6**) mediated by the acid H-X (Scheme 5, version A) or via a (monomolecular) intramolecular proton exchange via a three-ring proton σ-complex (Scheme 5, version B). Therefore, statements on the reactivity of these compounds are only correctly possible if these circumstances are taken into account. However, no detailed studies in this respect have been undertaken so far.

#### 3.2.2. Alkyl or Aryl Group Migration

**Scheme 6:**
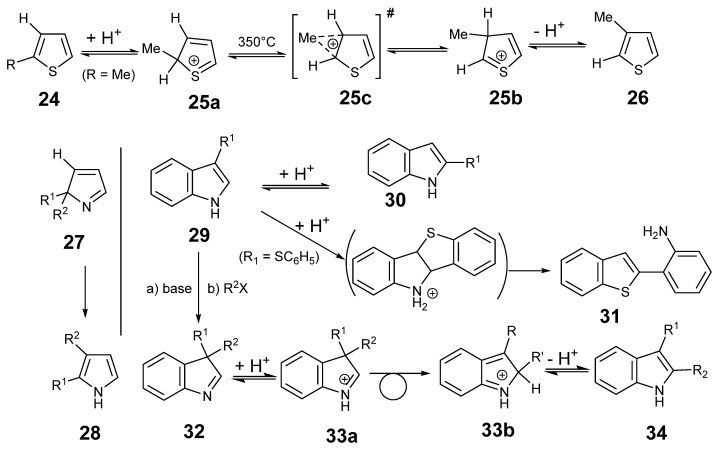
Mechanism of isomerisation reactions for some heteroaromatics initiated with Brønsted acids.

Instead of the proton migration in the proton adducts **11** discussed above, an alkyl or aryl group migration can also occur. However, this reaction requires usually higher temperatures. Thus, 2-methylthiophene (**24**) and its 3-methyl isomer (**26**) can be transformed into each other under the influence of acidic zeolites by heating to temperatures of about 350 °C via intermediated cations **25a**–**25c** [58,59,60,61] (Scheme 6).

Alkyl group migration was also observed for some special 2*H*-pyrrole derivatives (**27**) with alkenyl groups at C2 at temperatures > 115 °C. In this case, however, the mechanism probably involves allylic rearrangement and not adducts to form the isomeric 1*H*-pyrroles **28** [62].

A migration of alkyl and aryl groups also occur in the indole series. Thus, a variety of 3-subtituted indole derivatives, such as 3-phenyl-, 3-benzyl-, 3-methyl- and 3-*tert*-butylindole (**29**) were transformed into their corresponding 2-substituted derivatives **30** through treatment with TFS or, better, with polyphosphoric acid at 100 °C [45]. A similar reaction occurred with 3-phenylindole by heating with ZnCl_2_ at 170 °C to yield the 2-phenyl isomer **30** (R^1^ = Ph) [63].

The transformation of 3-phenythioindole (**29**, R^1^ = SC_6_H_5_) into 2-phenylthioindole (**30**, R^1^ = SC_6_H_5_) as an example of migration of a heteroatom-containing group was observed for treatment with polyphosphoric acid at about 100 °C (Scheme 6). Thereby, the simultaneous formation of 2-anilinobenzthiophene (**31**) was observed as result of a ring-opening/ring closure reaction [64].

As further examples of group migrations, the transformation of 3,3-disubstituted 3*H*-indoles **32** into 2,3-disubstituted 1*H*-indoles **34** was documented (Scheme 6) [65,66]. This process occurred by heating of the starting compounds in ethanolic 6 N HCl for 15 min. However, the rearrangement was accompanied by oligomerisation and cyclotrimerisation reactions in some cases [67].

**Scheme 7:**
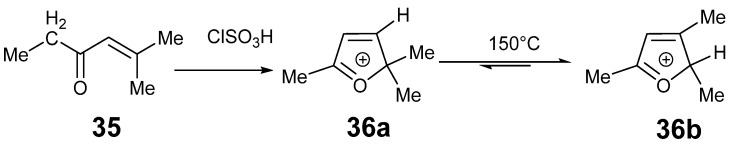
Transformation route of **35** into **36b**.

Alkyl group migration can also occur at some furan derivatives. For example, the trimethyl-substituted furylium cation (**36a**), which is formed through treatment of mesityl oxide (**35**) with chlorosulphonic acid, is transformed into the isomeric furylium cation **36b** following a methyl transfer at 150 °C [68] (Scheme 7).

**Scheme 8:**
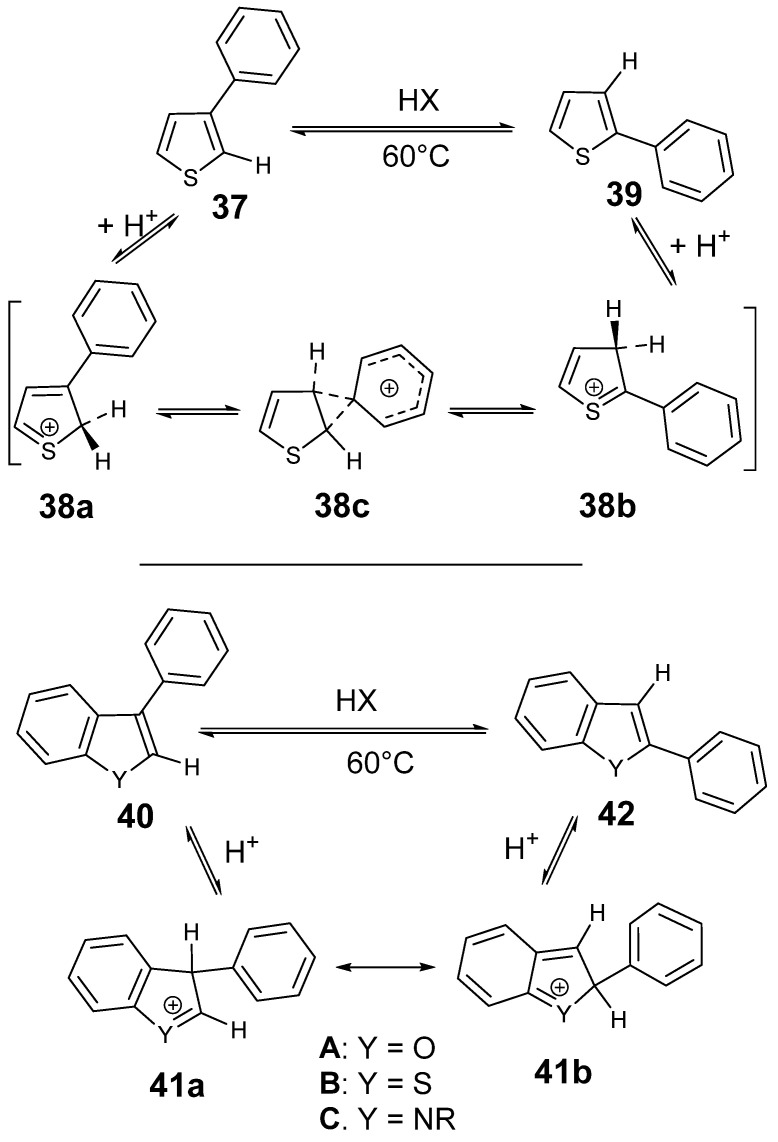
Isomerisation mechanism at some phenyl-substituted heteroaromatics catalysed with Brønsted acids.

Treatment of 3-phenylthiophene **37** with TFS or certain Lewis acids, such as heavy metal triflates at 60 °C, causes phenyl migration to the C2 position until an equilibrium between both phenyl derivatives **37** and **39** is established. Cationic species of the structure **38a**, **38b** and **38c** have been assumed as intermediates (Scheme 8) [69].

This type of reaction was referred to as an “aryl dance” and has some analogies in the series of different aromatic compounds [69] as well as with halo-substituted compounds, which can undergo a so-called halogen dance, but in this case, it is catalysed by bases [70].

In analogy to the indole derivatives **29** discussed earlier (Scheme 6), some benzo[*b*]thiophene **40B** and benzo[*b*]furan derivatives **40A** can react. They were transformed into their isomeric compounds **42A** and **42B** via treatment with Brønsted (or Lewis acids) at elevated temperatures. Proton adducts of the general structures **41a** and **41b** were assumed as intermediates. [69].

It is worth mentioning that an aryl migration can alternatively be initiated by certain heavy metals, such as Pd [71]. However, in this case, another mechanism exists involving hetaryl-Pd species that undergo Pd-migration.

**Scheme 9:**
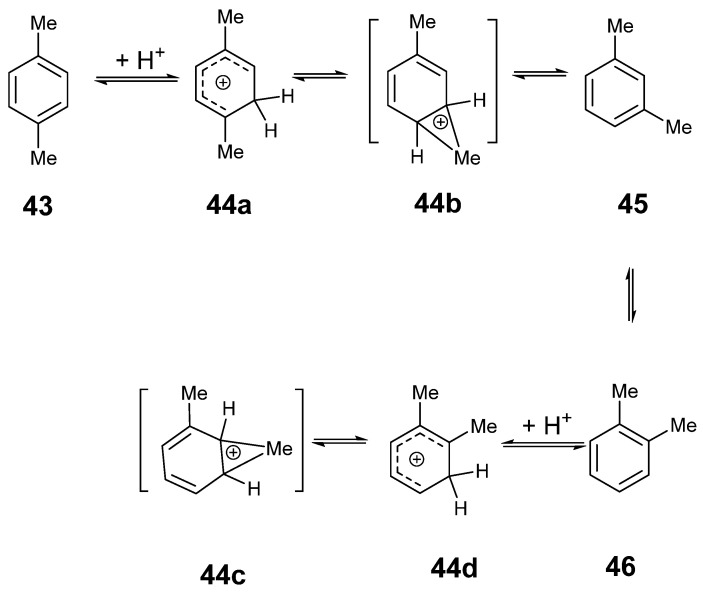
Brønsted-catalysed isomerisation at xylenes.

The isomerisation reactions for heterocyclic compounds regarded here have some analogies in the series of aromatic compounds. For example, *p*-xylene (**43**) and *o*-xylene (**46**) are transformed into their meta isomer **45** through treatment with TFS [56]. Under similar conditions, ethyl benzene yielded a mixture of benzene and *m*-diethylbenzene. All these reactions occur even at room temperature and run via cationic intermediates such as **44a**–**44d** [57] (Scheme 9).

#### 3.2.3. H/D-Exchange at (Hetero)aromatic Compounds

Detection of proton adducts **11a** and **11b** (Scheme 3) in reactions of Brønsted acids with heteroaromatics **6** can be challenging in particular if they occur only to a low extent (Scheme 3). In such cases, the use of deuterated acids D-X instead if H-X is very helpful because H/D exchange takes place and can easily be observed. The resulting deuterated compounds **13** and **14** are formed via the corresponding adducts **12** unambiguously and can be reliably analysed through NMR spectroscopy.

All the heterocyclic compounds **6A**–**6C** were transformed into the corresponding deuterated derivatives **14A**–**14C** via treatment with deuterated acids (Scheme 3). For example, thiophene (**6B**) was converted into its perdeuterated compound **14B** via treatment with deuterated sulphuric acid at RT for 4 days [72], via interaction with deuterated polyphosphoric acid (prepared through treatment of P_2_O_5_ with D_2_O) [73] or via treatment with deuterated acetic acid [72]. Analogously, pyrrole (**6C**) was transformed into its per-deuterated pyrrole **14C** through interaction with deuterated acetic acid [74], with deuterated methanol in the presence of catalytic amounts of HClO_4_ [75] or with DBr, generated through treatment of CD_3_OD with prenyl bromides [76]. It is worth mentioning that the deuteration of pyrrole and its *N*-substituted derivatives with suitable deuterating reagents has to be performed under weakly acidic conditions, because an oligomerisation of the starting compounds occurs otherwise. This oligomerisation can also be prevented if very strong acids, such as deuterated trifluoromethanesulfonic acid, are used. In this case, the concentration of the corresponding non-protonated pyrrole as a precondition for an oligomerisation is too small. The same statement is valid for the reaction of deuterated Brønsted acids with thiophenes. By using relative weak Brønsted acids, an oligomerisation is initiated instead of an H/D exchange (v.i. Section 3.2.4).

**Scheme 10:**
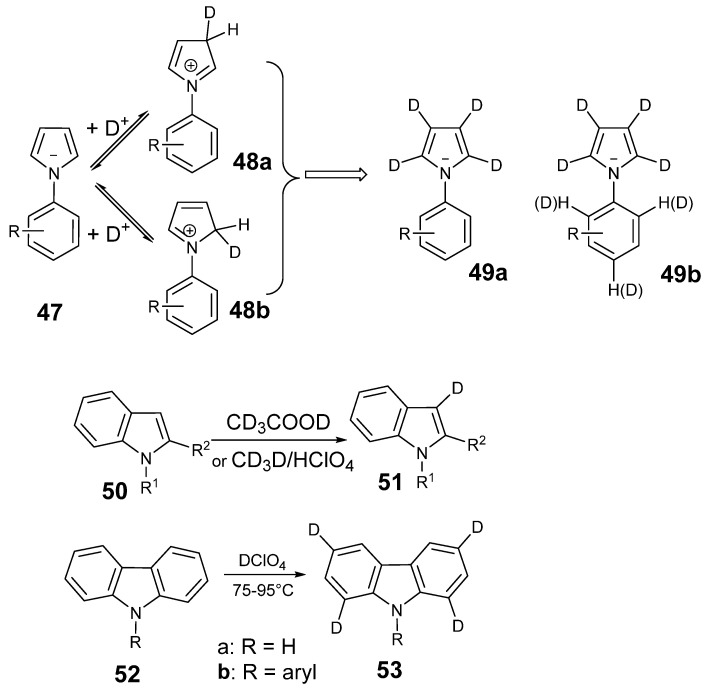
Reaction of *N*-phenylpyrrole (**47**), indoles **50** and carbazoles **52** with deuterated Brønsted acids.

An interesting phenomenon is observed in H/D exchange reactions with *N*-arylpyrroles **47** when treated with deuterated TFS [31]. Mixtures of analogously deuterated compounds **49a** and pyrroles **49b** with partly deuterated phenyl-substituent are generated (Scheme 10).

For furan (**6A**), an acid-catalysed D/H-exchange occurs in methanolic sulphuric acid at 20 °C [77]. All experiments with deuterated acids indicate an H/D exchange at both the alpha and beta positions of the heterocycles **6A** and indicate, therefore, the intermediate formation of the cationic compounds **11a** and **11b** as well as **12a** and **12b** (Scheme 3). In some cases, the relative extent of this H/D exchange has been estimated by means of usual ^1^H NMR measurements [76].

The relative D/H exchange rates between the C2 and C3 positions of deuterated thiophene, pyrrole and furan as well as selenophene were estimated with kinetic measurements [77]. Thereby, it was found that the exchange rate of thiophene (**6B**) at C2 is much higher than at the C3 position. In contrast, for furan (**6A**), these rates are nearly equal for both positions, but somehow inaccurate because of interfering ring splitting.

H/D exchange reactions have also been observed in the series of indoles (Scheme 10). Thus, the treatment of indole derivatives (**50**) with CD_3_COOD or DBr results in deuterated indoles **51** via deuteration of position C3 [76]. H/D exchange at C2, C4 and C6 was observed at some indole derivatives, such as melatonin and serotonin, with CD_3_OD in presence of 5 mol% HClO_4_ [76].

As mentioned above, an H/D exchange also occurs at carbazole (**52a**) under these conditions (Scheme 10). Thereby, a deuterated carbazole (**53a**) was obtained in a yield of 95% with deuterium contents of 43% (at C1 and C8) and 78% (at C3 and C6). This result indicates that these positions in 9*H*-carbazole (**52a**) can be protonated with acids HX in which tautomeric 1*H-* or 3*H-*carbazole species occur as intermediates (Scheme 4) [52].

An H/D exchange also takes place at benzo[*b*]thiophene (**16B**) at position 2 and benzo[*b*]furan (**16A**) at positions 2 and 3 if these compounds are treated with D_2_O in DMSO-*d*_6_ in the presence of Ag_2_CO_3_ and triarylphosphine as catalysts (Scheme 3) [78]. The authors assume that the reaction runs on a complex of the benzo-analogue of the general structure **7a** with E = Ag-triflate. An analogous H/D exchange occurs if the same reaction is performed in CH_3_OD [78].

H/D exchange at benzo[*b*]thiophene (**16B**) can also be performed with phosphine ligated Ag-carboxylates in the presence of Ph_3_P [79]. Thereby, the starting compound is transformed into its 2-deuterated derivative in 60% yield during 16 h.

Other possibilities to perform an H/D exchange at benzo[*b*]thiophene (**16B**) are provided by RuCl_3_, FeCl_3_ or CrCl_3_ dissolved in D_2_O at elevated temperatures. D^+^[M(D_2_O)_5_OD]^2+^ are assumed as the reactive species [80].

In this context, it should be mentioned that the formation of proton adducts was also observed with *N,N*-diaryl-substituted 2-aminothiophenes **54** [81]. These compounds were protonated through treatment with TFS not only at their *N*-atom but also at C5.

**Scheme 11:**
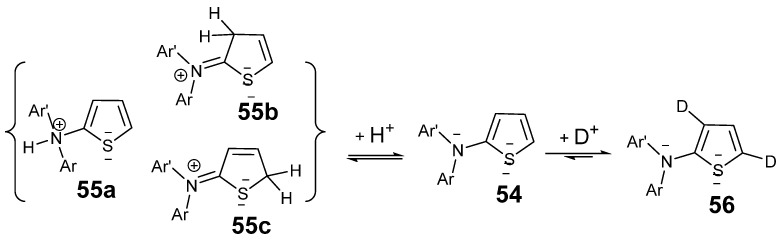
Reaction of 2-(*N,N*-diarylamino)thiophenes with Brønsted acids and their deuterated derivatives.

With TFA-d, deuteration at C3 and C5 occurs to afford the bis-deuterated compounds **56**. This fact allows the conclusion that proton adducts **55b** and **55c** form in concurrence to the formation of the generally expected ammonium salt structure **55a** when non-deuterated TFS is used (Scheme 11). This was verified by the formation of the 3,5-dideuterated product upon treatment with CF_3_COOD [81].

In this context, it is remarkable that naphthyl-substituted 2-aminothiophenes (**55** with Ar = 1- or 2-C_10_H_7_) undergo not only H/D exchange at their thiophene moieties but also partially at the naphthalene groups. This reaction has some analogy to the reaction of *N*-naphthyl-substituted diphenylamines, where H/D exchange was observed at the naphthyl moieties too [52].

#### 3.2.4. Brønsted Acid-Catalysed Oligomerisation of (Hetero)aromatic Compounds

In the examples discussed so far, reaction of (hetero)aromatic compounds **6** and their benzo-condensed derivatives **16** and **21** with strong acids stopped at the protonation (or deuteration) of their rings. Nevertheless, certain subsequent reactions, such as dimerisations, olgomerisations and isomerisations, are also possible as repeatedly documented in the literature.

**Scheme 12:**
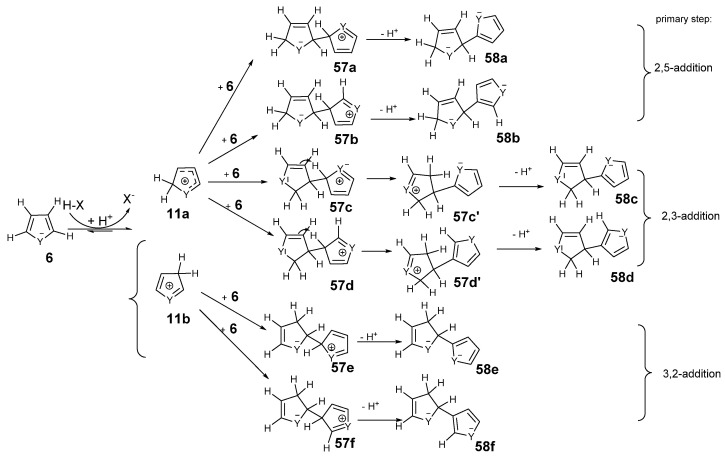
Mechanism of Brønsted acid-catalysed dimerisation of heteroaromatics **6**.

Obviously, all these reactions are initiated by the formation of the proton adducts **11a** and/or **11b** (Scheme 3), which exhibit significant electrophilic character. This property enables these compounds to react with their non-protonated parent compounds **6** as species with decidedly nucleophilic character (Scheme 12). Thereby, six different products **58a**–**58f** can be formed primarily. Their structures depend on the ring positions involved in the coupling reactions. Thus, by coupling of the proton adduct **11a** at C5 with the parent compound **6** at C2 or C3, the dimers **58a** and **58b** are formed via the corresponding primarily generated adducts **57a** and **57b**, respectively, in the course of a 2,5-addition at the heterocyclic starting compound **6**.

Analogously, by coupling of the proton adduct **11a** at C3 with the parent compound **6** at C2 or C3, the dimers **58c** and **58d** are formed via the corresponding primary adducts **57c** and **57d** or their isomeric compounds **57c’** and **57d’**, respectively, in the course of a 2,3-addition. Finally, dimers **58e** and **58f** are formed by coupling of the proton adduct **11b** at C2 with the parent compound **6** at C2 or C3 via the corresponding primary adducts **57e** and **57f**, respectively, in the course of a 3,2-addition.

In special cases, the dimers **58a**–**58f** are able to react with a further equivalent of the appropriate proton adducts **11a** or **11b** under formation of the corresponding trimers. Their structure depends again on the ring positions involved in the coupling reactions. Hence, a large variety of different products can be formed through coupling reactions starting from the simple aromatic compounds **6** treated with Brønsted acids.

The variability of these dimerisation reactions is somewhat restricted during reaction of the benzo condensed heteroaromatic compounds of the general structure **16** with their corresponding proton adducts **17**, because only a 2,3- or 3,2-addition can occur. In fact, only one of them will generally appear in a reaction of a heteroaromatic compound **16** with its corresponding proton adduct **17a** and **17b** (Scheme 3).

**Scheme 13:**
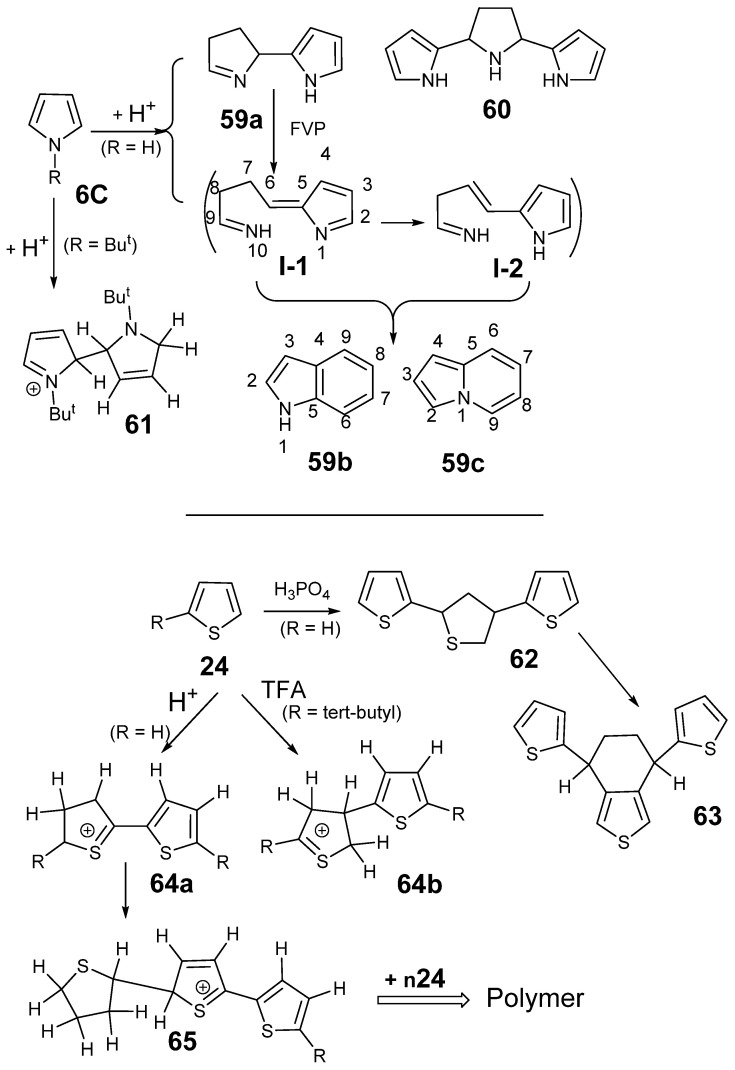
Mechanism of the Brønsted acid-catalysed oligomerisation of pyrrole (**6C**) and thiophenes (**24**).

However, such restriction does not exist with simple heteroaromatic compounds non-substituted at their heteroaromatic core. Thus, pyrrole (**6C**) is transformed into a trimer of the structure **60** besides some polymeric compounds via treatment with simple mineral acids, such as with aqueous hydrochloric acid for 30 s [82,83]. Cations **11a** and **11b** (Scheme 12) will primarily form when reacting with unprotonated pyrrole (**6c**). However, only the dimer **59a** was obtained when pyrrole was treated with aqueous HCl at 0 °C for 5 s. At higher temperature, under flash vacuum pyrolysis, this compound could be transformed into indole (**59b**) and indolizine (**59c**) [84] (Scheme 13).

A similar reaction occurs with several *C*-alkyl and *C*-phenylpyrroles, which are transformed into corresponding *C*-substituted dimers [85] through treatment with aqueous HCl, or, under slightly changed conditions (heating of the appropriate pyrrole with acetic acid), into the corresponding *C*-substituted indoles [67]. Obviously, the corresponding proton adducts and dimers are the necessary intermediates for this transformation.

As indicated with ^1^H NMR measurements, dimers and polymers are also formed when *N*-arylpyrroles (**47**) are treated with TFA [31], whereas proton adducts as H-analogues of **48b** appear in the presence of TFS.

Analogously, *N*-(*tert*-butyl)pyrrole **6C** (R = *t*-Bu) was transformed into dimer **61** through treatment with TFA. Its structure was elucidated via ^1^H NMR measurements (Scheme 13) [31]. However, a stable proton adduct of the general structure **11Cb** (Y = N-*t*-Bu) was formed with treatment of this *N*-substituted pyrrole with TFS.

Similar oligomerisation reactions like in pyrroles were observed in the thiophene series. Thus, the treatment of the parent thiophene **24** (R = H) with phosphoric acid gave rise to a trimer of the structure **62** [86,87] (Scheme 13). Remarkably, the structure of this thiophene trimer **62** differs from the pyrrole trimer **60** with respect to the linking positions of the heterocyclic rings.

Through treatment of thiophene **24** (R = H) with trichloroacetic acid or TFA, a polymerization occurs. Thereby, polymers with ca. fifteen thiophene and tetrahydrothiophene units are formed [88,89].

However, the corresponding solid trimers were obtained through treatment of alkylthiophenes **24** with phosphoric acid [86,87]. The interaction of these thiophene derivatives with acid-activated clay at 150–170 °C resulted in dimers and trimers with cationic structures **64a**, **64b** and **65** as well as polymers [90].

A similar result was obtained through treatment of some thiophene derivatives, such as 2- and 3-methylthiophene, with TFA [12]. However, no detailed information on the structures of the oligomeric products could be derived from the ^1^H NMR spectra. On the other hand, the ^1^H NMR spectrum of 2-methylmercaptothiophene **24** (R = MeS) in TFA shows characteristic signals in the region between 3.0 and 5.0 unambiguously verifying the formation of a dimer of the structure **64b** with R = MeS [12].

**Scheme 14:**
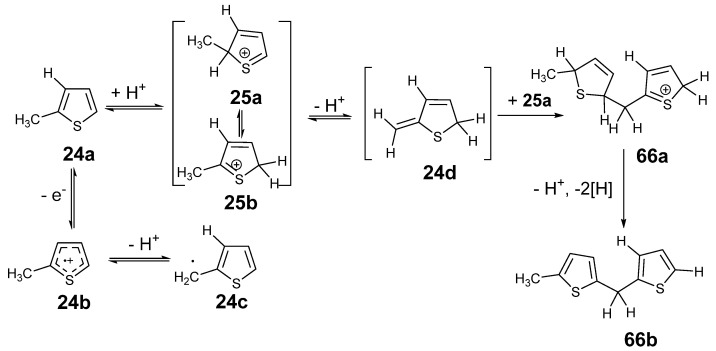
Mechanism of the Brønsted acid-catalysed dimerisation of 2-methylthiophene (**24a**).

Another result was obtained if 2- or 3-methylthiophene was treated with zeolites at higher temperature [61]. Thereby, not only a migration of the methyl groups as discussed in Scheme 6 can occur but also a dimerisation under incorporation of the methyl groups arises. The mechanism depicted in Scheme 14 can be assumed. 2-Methylene-2,5-dihydrothiophene (**24d**) can be assumed as an intermediate in this methylene bridging. An alternative mechanism also seems possible involving the primary formation of the radical cation **24b** that is transformed into the radical **24c** and subsequently attacks a parent thiophene under formation of a dimeric compound (Scheme 14).

This type of dimerisation was also observed for the 2- and 3-methyl-substituted benzo[*b*]thiophenes [60]. Here, the two benzo[*b*]thiophene moieties were connected at their heterocyclic or carbocyclic moiety via the methyl groups.

**Scheme 15:**
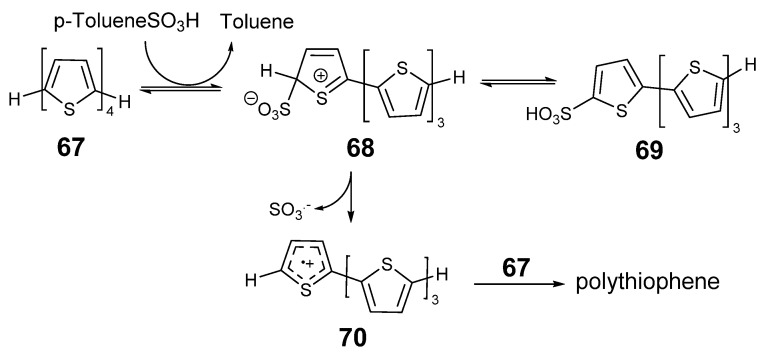
Mechanism of *p*-toluenesulphonic acid initiated polymerisation of tetrathiophene **67**.

The polymerisation of tetrathiophene (**67**) can be accomplished by using *para*-toluenesulphonic acid at about 100 °C in the gas phase [91] (Scheme 15). It was assumed that under this condition, the *p*-toluenesulphonate can split into toluene and SO_3_ that generates the corresponding adduct **68**. This adduct can be either transformed into the corresponding thiophene-2-sulphonic acid derivative **69** [91] or splits into the radical anion (SO_3_^−^) and the thiophene radical cation **70**, which start a polymerisation by reaction with the starting thiophene oligomer (**67**). Under the same condition, terthiophene can also be polymerised [92]**.**

**Scheme 16:**
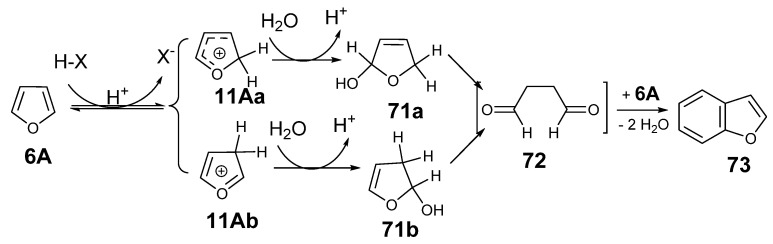
Mechanism of the Bronsted acid-catalysed ring opening of furane (**6A**) and the subsequent recyclisation of the intermediately formed succinic aldehyde (**72**) to benzofurane (**73**).

In contrast to the oligomerisation and polymerization reactions of pyrrole and thiophene, a slightly different behaviour was observed with furan (**6A**) when treated with Brønsted acids. By using aqueous acids, this compound and some of its 2,5-dialkyl-substituted derivatives underwent a facile ring opening reaction resulting in the corresponding 1,4-dicarbonyl compounds, e.g., **72** (Scheme 16) [93]. At a higher temperature, furan (**6A**) was transformed into benzofuran (**73**) as well as into polymeric products through treatment with mineral acids or zeolites [94,95,96,97,98]. In the first step of this transformation, a ring opening occurs initiated by the formation of the corresponding proton adducts **11Aa** or **11Ab**. The resulting succinaldehyde (**72**) undergoes a cycloaddition with the parent furan **6A**. It is worth mentioning, that the ring-opening failed when furans were treated with super acids [16,17,68].

Acid-catalysed dimerisation reactions are also reported for benzofurans [99], benzothiophene [100] and indole [101,102,103,104]. The latter can also form triindol [103] through treatment with phosphoric acid [105]. Indole and 3-substituted indole produce mixed dimers and trimers upon treatment with gaseous HCl at low temperatures [106].

### 3.3. Formation and Reactions of Lewis Acid Adducts with Simple Heteroaromatic Compounds

**Scheme 17:**
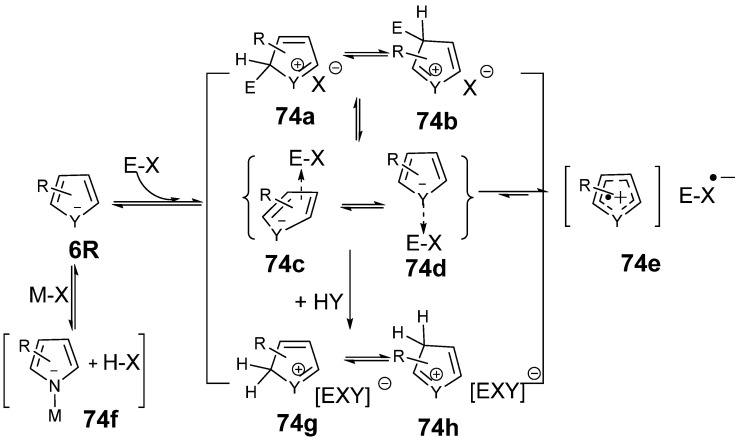
Product formation in the reaction of heteroaromatics **6** with Lewis acids E-X.

Besides the addition of protons to the heterocyclic compounds **6** initiated with Brønsted acids, which gives rise to the formation of proton adducts of the structure **11a** or **11b**, there are many hints for the formation of addition products of Lewis acids E-X with heterocyclic compounds **6** or some of their benzo-condensed derivatives **16**. However, in most cases, the structures of such addition products are unknown yet, but they were usually indicated by reactions initiated by the Lewis acids added to the heteroaromatic starting compounds. Nevertheless, a priori, these addition compounds can exhibit different structures. Thus, they can have the π-complex structure **74c**, a σ-complex skeleton **74a** or **74b,** in which the Lewis acid is added at one of the C-atom, or form structures **74d,** wherein E-X coordinates to the hetero atom. If the heterocycle **6R** reacts with a sterically congested Lewis acid, e.g., B(C_6_F_5_)_3_, so called frustrated Lewis pairs are formed maintaining both the Lewis acidity of the Lewis acid and the Lewis basicity of the heterocycle. Such compounds have gained application for introducing substituents into the heterocycle **6R** as well as in catalysed hydrogenations [107,108,109]. Moreover, the addition compounds of heteroaromatics with Lewis acids can be radical cations **74e** if they are formed in the course of an electron transfer between the starting heterocyclic compound **6** and the Lewis acid E-X. Last, but not least, structures **74f** are possible, if *NH*-substituted pyrroles are used (Scheme 17).

Furthermore, a corresponding Brønsted acid H^+^[EXY]^−^ can be formed if another weak Brønsted acid H-Y, such as water, which is unable to react with the heteroaromatic compounds **6**, is present in the reaction mixture. This creates a cationic Brønsted adduct **74g** or **74h** enabling various subsequent reactions of the heterocyclic compound **6**. Taking AlCl_3_ as an example of a typical Lewis acid, the formation of Brønsted acids via its reaction with water has been examined in more detail. Very strong Brønsted acids of the general formula H^+^[(AlCl_3_)_n_OH]^−^ with strongly negative pKa values were formed [110].

As can be derived from the experimental data, the structures of the addition products formed through the reaction of Lewis acids with heteroaromatic compounds **6** or their benzo-condensed derivatives **16** depend not only to a certain extent on the structure of the corresponding heterocyclic compound, but also significantly on the chemical nature of the Lewis acid, especially on its hardness.

Besides AlBr_3_, the following Lewis acids arranged by their strength according to the scale of Kobayashi et al. [111] were used in typical acid base reactions with heteroaromatics: TiCl_4_ > AlCl_3_~SnCl_4_ > SbCl_5_ > SnCl_2_ > ZnCl_2_. Furthermore, also FeCl_3_, CuCl_2_ and HgX_2_ were applied but react in different ways, as shown later (v.i.)

**Scheme 18:**
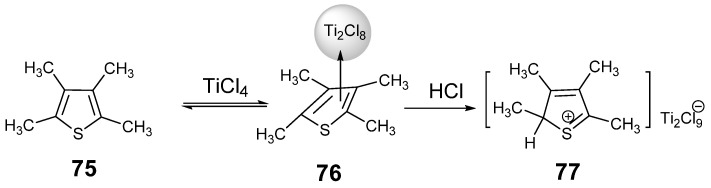
Structure and transformation of the complex **76** formed via reaction of tetramethylthiophene (**75**) with TiCl_4_.

Despite many hints to the formation of adducts between the heteroaromatic compounds **6** and **16** and different types of Lewis acids, there are only few examples in the literature documenting the structure of such addition products. In most cases, the formation of these adducts was deduced from the reactions initiated by treatment of the heteroaromatic compounds with the respective Lewis acid. Nevertheless, few Lewis acid adducts have been unambiguously indicated. For example, addition of TiCl_4_ to a solution of tetramethylthiophene (**75**) in CH_2_Cl_2_ resulted in a red-coloured product. Because this compound can be transformed into the proton adduct **77** through treatment with gaseous HCl, the structure **76** for this primary product has been stated (Scheme 18) [3].

**Scheme 19:**
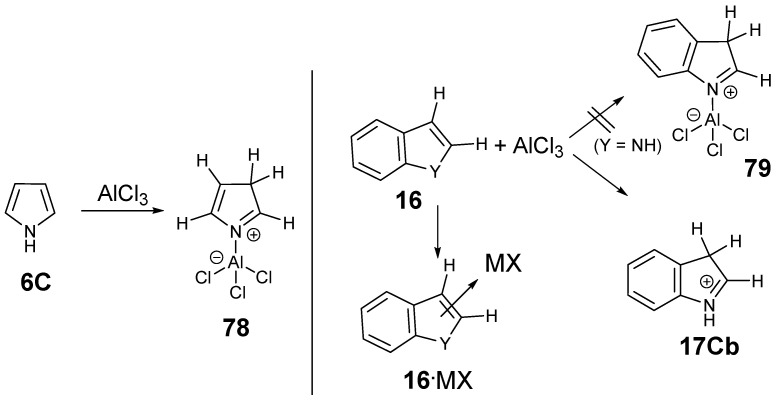
Structures of products formed via reaction of pyrrole (**6C**) and heteroaromatics **16** with AlCl_3_.

Similarly, pyrrole (**6C**) forms a zwitterionic complex **78** with AlCl_3_ in an ionic liquid as elucidated with NMR spectroscopy (Scheme 19) [112].

However, this finding contradicts with results found through treatment of pyrrole (**6C**) and indole (**16C**) with AlCl_3_ in CD_3_NO_2_. Thereby, the formation of corresponding proton adducts **11Cb** and **17Cb** were indicated and their structure was confirmed with NMR spectral data [113]. Thus, in the ^1^H NMR spectrum of the product resulting from treatment of pyrrole (**6C**) with AlCl_3_ in CD_3_NO_2_, five proton signals at 5.2, 7.0, 8.3, 9.2 and 11.42 ppm with an intensity ratio of 2:1:1:1:1 were recorded. Whereas the first mentioned signal stems from the CH_2_ moiety in the compound **11Cb**, the last one, which occurs as characteristic triplet, stems from the proton at the *N*-atom. Obviously, this species owes its formation to the interaction with the Brønsted acid H^+^[(AlCl_3_)_n_OH]^−^ as mentioned above [112].

Analogous results were obtained with treatment of *N*-arylpyrroles **47** and *N*-arylindoles **50** (R1 = aryl) with AlCl_3_ in nitromethane. Again, the formation of the corresponding proton adducts instead of the corresponding Lewis acid adducts was observed and their structures were confirmed with ^1^H NMR measurements [113].

In addition to the studies on the elucidation of the chemical structures of complexes formed through interaction of the heteroarenes with Lewis acids discussed above (Scheme 17) there are a lot of studies on the role of these complexes in oligomerisation or polymerization of the starting materials. Depending on the oxidation strength of the Lewis acids used, polymers with full-conjugated structures or with structures containing non-aromatic moieties in their molecular frameworks can result. Whereas the first-mentioned type of polymers stemming obviously from the reaction of the heteroarenes with FeCl_3_ (Fe^3+^/Fe^2+^ = +0.77 V), CuCl_2_ (Cu^2+^/Cu^+^ = +0.16 V) or SnCl_4_ (Sn^4+^/Sn^2+^ = 0.14 V) via oxidation, the other ones derive from the reaction with a Lewis acid, e.g., AlCl_3_ (Al^3+^/Al = −1.66 V). However, only in certain cases do detailed studies exist on the formation mechanism of the polymers. A priori, the formation of the polymers with a fully conjugated π-system can occur in two ways, either primary formation of a radical cation **74e** (Scheme 17) takes place that initiates the subsequent reactions by its addition to a parent heteroarene [114,115,116,117] or, alternatively, later oxidation of the polymer formed through a primary addition of a Lewis acid complex **74a** or **74b** to the parent heteroarene occurs in analogy to a mechanism depicted in Scheme 12 [89,118,119].

**Scheme 20:**
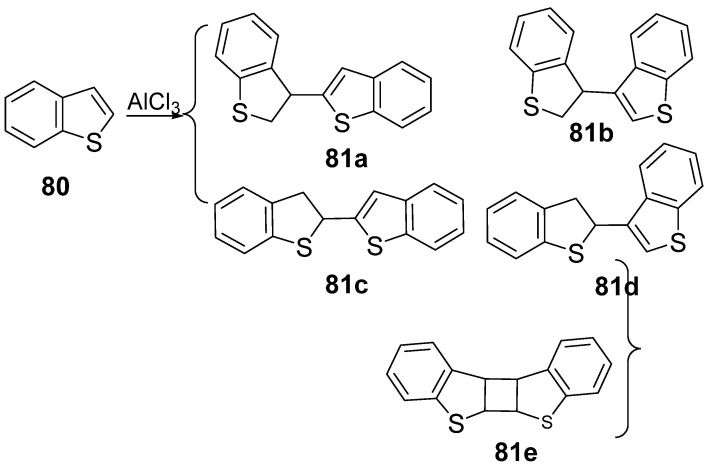
Products generated through reaction of benzothiophene **80** with AlCl_3._

An oligomerisation also happens through treatment of benzo[*b*]thiophene (**80**) with AlCl_3_ [100] (Scheme 20) resulting in dimers **81a** and **81b**. In contrast, an arylation at the C2- and/or C3-position of **80** rather than an oligomerisation takes place when aromatic solvents are used. Treatment of benzo[*b*]thiophene **80** with Al_2_(SO_4_)_3_ in supercritical CO_2_ results in the formation of the dimers **81a** and **81b** and three further dimers **81c**–**81e** [120].

Another interesting result was obtained via treatment of indole **16C** with SnCl_4_. After addition of this Lewis acid to a solution of indole **16C**, a product of the formula (**16C**)_2_·SnCl_4_ precipitated from which indole **16C** could be re-isolated after treatment with ammonia [121,122]. Analogous results were achieved with SnBr_4_, TiCl_4_ and AlBr_3_. In these cases, products of the composition (**16C**)_2_·SnBr_4_, (**16C**)_2_·TiCl_4_ and (**16C**)_2_·AlBr_3_, respectively, were obtained. Because the subsequent treatment of these compounds with ammonia regenerates the parent indole **16C**, the corresponding π-complexes seem to be formed. As shown with SnCl_4_, these adducts decompose into oligomers, thus they are intermediates in Lewis acid-catalysed oligomerisation/polymerisation [119]. It has to be mentioned that nitrogen-free heteroaromatics, e.g., thiophene [123,124,125] and furan [126] and their benzo-condensed derivatives do not form analogous complexes but structures similar to the complex **7a** or **7b**. When furan (**6A**) is treated with ZnCl_2_, TiCl_4_ or SnCl_4_, intermediate Lewis acid complexes **74** are assumed, which initiate its polymerisation [126].

In contrast to the results mentioned above, where treatment of the heteroaromatic compounds **6** and **16** with different Lewis acids gives rise to the formation of the corresponding Lewis acid adducts, the treatment of these heteroaromatics with FeCl_3_ and/or CuCl_2_ follows another course. Here, the Lewis acid does not react as such but as an oxidizing reagent primarily forming a radical cations **74e**. This reacts further with starting heteroaromatic compounds **6** to form oligomers or polymers. Since this type of reaction is out of the scope of our review, we just provide some hint to representative examples of these reactions. They can be found in the furan series **6A** in references [127,128,129], in the thiophene series **6B** in references [130] and in the pyrrole series **6C** in references [119,131,132,133,134].

**Scheme 21:**
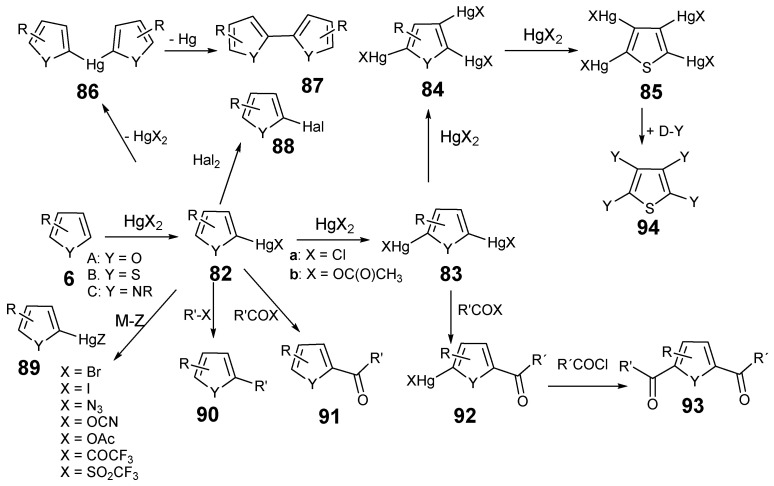
Products generated through reaction of the heteroaromatics **6** with certain Hg(II) salts.

Interesting results were obtained through reaction of furan **6A**, thiophene **6B** and pyrrole **6C** as well as their benzo condensed derivatives with certain mercury(II) salts HgX_2_ wherein X = Cl, OAc. Thus, treatment of furan **6A** or thiophene **6B** with mercury chloride (HgCl_2_) in aqueous solution and in the presence of sodium acetate leads to mercury substitution products **82**–**85** as solid and air- and water-stable compounds in a very straight forward way (Scheme 21) [135,136,137,138,139,140]. These transformations are very remarkable because they represent direct metalation reactions via *H*-metal exchange that usually can only be achieved with very reactive metals or organometallics, such as RLi under inert conditions [141]. In the case of mercury-substituted heteroarenes, water and polar solvents can mostly be used, whereas inert conditions are not necessary. An older comprehensive review on the chemistry of thiophene mercury compounds was provided by Steinkopf in 1941, who studied these compounds extensively for a longer period [142].

In contrast to the formation of the mercury compounds **82**–**85** obtained from furan (**6A**) or thiophene (**6B**), an adduct of the general formula (**6C**)_2_Hg^.^(HgCl_2_)_4_ was obtained when pyrrole (**6C**) was treated with HgCl_2_ in acetic acid [143]. Performing the reaction of pyrrole with HgCl_2_ in water gave rise to partial hydrolysis resulting in a presumed mixed complex of (**6C**)(HgCl)_2_HgClO. This forms polypyrrole single- and double-shelled hollow nanospheres in the presence of oxidizing reagents [144].

**Scheme 22:**
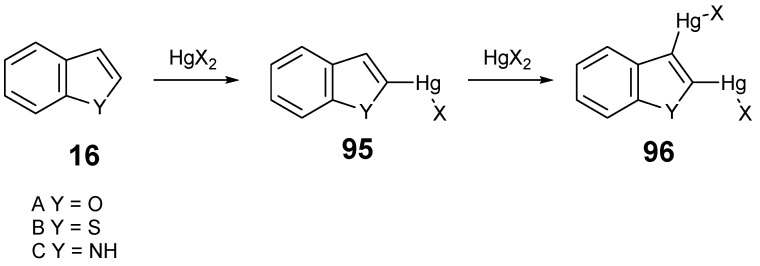
Reaction of benzofuran, benzothiophene and indole with Hg(II) salts.

The mercuration reaction was also performed with benzo[*b*]furans (**16A**) [145] and benzo[*b*]thiophenes (**16B**) [146] using Hg(OAc)_2_ or HgCl_2_ (Scheme 22). While the mercuration of benzofuran (**16A**) yields the mono-mercurated compound **95A** (X = Cl) through treatment with HgCl_2_ [145] in hot ethanol, the reaction continues to the bis-mercury compound **96B** if benzo[*b*]thiophene (**16B**) is treated under these conditions [146]. The reaction stops at the mono-mercurated benzo[*b*]thiophenes **95B** (X = OAc), however, when HgOAc_2_ in boiling methanol is used (Scheme 22) [147,148].

The treatment of indole (**16C**) with Hg(OAc)_2_ in ethanol gives rise to the corresponding 2,3-di(acetylmercury)indole [149]. By working in Ac_2_O, an acetylmercury group and an acetyl moiety are introduced in position 3 and at the *N*-atom (Y = NAc), respectively [150].

1-Methylindole was 3-mono or 2,3-bis-mercurated with HgCl_2_ or Hg(OAc)_2_, respectively, in ethanol or ethanol/diethyl ether [151,152].

Alternatively, mercurated heterocycles **82** and **83** can also be prepared through reaction of metallated heterocycles, e.g., of lithiated species [153,154] or boron derivatives [155], with mercury chloride under inert conditions.

Mercurated heterocycles **82** and **83** can be used as educts for the synthesis of a variety of products. Thus, the mercurated compounds **82** can undergo exchange reactions at their HgX moieties yielding different mercury-substituted compounds **89** when treated with alkali halides or pseudohalogenides as well as with silver acetate or silver triflate [156]. Moreover, the mercurated compounds **82** and **83** can be applied as educts for the synthesis of mercury-free products, such as for the synthesis of alkyl-substituted compounds **90** [157] and acyl substituted products **91**–**93**. For example, alkyl-substituted compounds **90** (R’ = Alkyl) result from the reaction of **82** with MeRh reagent [158], with *tert-*Bu-Cl [157] or with vinylcarbonyl compounds [159]. The acylated derivatives **91**–**93** are generated through treatment of the appropriate mercury compounds **82** or **83** with certain acylating reagents [135,142]. The reaction of the tetramercury compound **85B** with D_2_O or TCl allows the synthesis of the perdeuterated or pertritiated thiophene derivate **94A** (Y = D) [160] or **94** (Y = T) [161], respectively. The iodo compound **88** (Hal = I) was obtained through reaction of compound **82A** with KI_3_ [162] while the corresponding chloro products **88** (Hal = Cl) were produced with disulphur dichloride [162].

Mercurated heterocycles **82** can be transformed into dihetaryl mercurials **86** in a so-called symmetrisation reaction by heating in an appropriate solvent [156,163,164,165], with basic aluminium oxide in toluene at room temperature [166] or in the presence of sodium iodide [167]. An aryl–aryl coupling to mercury-free biheteroaryl **87** can be achieved through heavy metal catalysis [163,168].

**Scheme 23:**
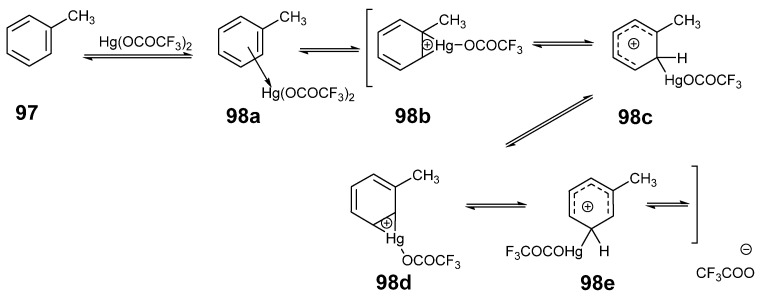
Products generated through reaction of toluene (**97**) with Hg(tfl)_2_.

Usually, the structures of the mercury compounds **82**–**85** were derived from their elemental analyses as well as from the verified structures of their reaction products. Nevertheless, for the compound **83** (X = tfl) and its bithiophene derivative, prepared through reaction of the corresponding parent thiophenes with Hg(tfl)_2_, X-ray analyses are known [130,169]. In these compounds, the metal organyl moieties are linked to the 2- and 5-positions of the thiophene core. This is in contrast to the compounds formed via reaction of Hg-II compounds with aromatic hydrocarbons. This reaction leads to compounds with variable structures **98a**–**98e** (Scheme 23), their proportion has been determined through subsequent reaction of the primarily formed Hg-adduct with bromine and analysis of the resulting product mixture [170].

It is worth mentioning that special mercury compounds have been prepared in the aromatic series also through reaction of appropriate Hg(I)-compounds, such as of Hg_2_(AsF_6_)_2_, with aromatic hydrocarbons [127]. The structures of these products are similar to the ones of the compounds **98** with the change that instead of [Hg(tfl)]^+^ the cationic group [Hg(AsF_6_)]^+^ is present therein. Corresponding investigations are unknown with heteroaromatics.

In addition to the papers dealing with experimental studies on reactions of electron-rich heteroaromatics with Brønsted and Lewis acids considered here, there are also several publications on corresponding theoretical studies. For reactions with Brønsted acids, RHF/6-31G, MP2/6-31G and B3LYP/6-31G methods [171,172], the MINDO method [173] and ab-initio calculation [174] were successfully applied. The results of F. P. Colonna [175] on the interaction of mercuryl halides with furan and thiophene are, in particular, worth mentioning.

Finally, it should be noted that there are also extensive publications on the reactions of arenes with Brønstedt acids, which were summarized in Topics of Current Chemistry I–III by V. A. Barkhash [176], V. G. Shubin [177] and by V. A. Koptyug [55] and could help to understand the chemistry of heteroaromatics reviewed here.

## 4. Conclusions

A first review about reactions of basic electron-rich heteroaromatic compounds such as furan, thiophene and pyrrole and their benzo-condensed derivatives with Brønsted and Lewis acids is provided. At first glance, the reaction of these heteroaromatic compounds **6**, **16** and **21** with Brønsted acids seems to be a simple equilibrium reaction whose direction is only determined by the strength of the acid used and the position-specific proton affinity of the heteroaromatic compound. A detailed consideration reveals, however, that the aromatic π-systems of the heteroaromatic compounds are destroyed by the proton addition giving rise to compounds with a high electrophilicity. This initiates intramolecular proton or substituent migrations as well as ring opening and ring transformation reactions. Moreover, intermolecular reactions with the nucleophilic starting heterocycle, giving rise to the formation of appropriate dimers, oligomers or polymers, can also be induced. Whereas the chemical structures of the products arising through such intermolecular reactions are mostly insufficiently elucidated, the corresponding structures of the primary proton adducts can be resolved well thanks to the use of deuterated acids and the application of modern structural–analytical methods, especially ^1^H NMR spectroscopy.

However, this statement is nearly not valid for the Lewis acid adducts formed through the reaction of Lewis acids with the heteroaromatic compounds. Although these adducts initiate similar subsequent reactions as observed with the aforementioned proton adducts, there is only sparse information on the structure of the Lewis acid adducts primarily formed. A similar situation is found in reactions of heteroaromatic compounds with Hg(II) salts, where usually only elemental analysis studies exist, or the structure of the primary adducts is concluded from subsequent transformations. Although heteroaryl–mercury compounds are extremely easy to prepare, exhibit a robust stability and offer a large synthetic potential, their application is limited because of toxicity issues.

The field of heterocyclic chemistry described in this review traces back more than 100 years and is still in the focus of contemporary chemistry with applications in the laboratory and industry. It still offers interesting challenges for synthesis also including substituted heterocycles, mechanistic research, theoretical methods and analysis exploiting the modern facilities existing nowadays.

## Data Availability

Not applicable.
